# High Levels of Antibiotic Resistance in Isolates From Diseased Livestock

**DOI:** 10.3389/fvets.2021.652351

**Published:** 2021-04-01

**Authors:** Nurul Asyiqin Haulisah, Latiffah Hassan, Siti Khairani Bejo, Saleh Mohammed Jajere, Nur Indah Ahmad

**Affiliations:** Faculty of Veterinary Medicine, Universiti Putra Malaysia, Serdang, Malaysia

**Keywords:** antimicrobial resistance, livestock, cattle, pigs, clinical, *Escherichia coli*

## Abstract

Overuse of antimicrobials in livestock health and production beyond therapeutic needs has been highlighted in recent years as one of the major risk factors for the acceleration of antimicrobial resistance (AMR) of bacteria in both humans and animals. While there is an abundance of reports on AMR in clinical isolates from humans, information regarding the patterns of resistance in clinical isolates from animals is scarce. Hence, a situational analysis of AMR based on clinical isolates from a veterinary diagnostic laboratory was performed to examine the extent and patterns of resistance demonstrated by isolates from diseased food animals. Between 2015 and 2017, 241 cases of diseased livestock were received. Clinical specimens from ruminants (cattle, goats and sheep), and non-ruminants (pigs and chicken) were received for culture and sensitivity testing. A total of 701 isolates were recovered from these specimens. From ruminants, *Escherichia coli* (*n* = 77, 19.3%) predominated, followed by *Staphylococcus aureus* (*n* = 73, 18.3%). Antibiotic sensitivity testing (AST) revealed that *E. coli* resistance was highest for penicillin, streptomycin, and neomycin (77–93%). In addition, *S. aureus* was highly resistant to neomycin, followed by streptomycin and ampicillin (68–82%). More than 67% of *E. coli* isolates were multi-drug resistant (MDR) and only 2.6% were susceptible to all the tested antibiotics. Similarly, 65.6% of *S. aureus* isolates were MDR and only 5.5% were susceptible to all tested antibiotics. From non-ruminants, a total of 301 isolates were recovered. *Escherichia coli* (*n* = 108, 35.9%) and *Staphylococcus* spp. (*n* = 27, 9%) were the most frequent isolates obtained. For *E. coli*, the highest resistance was against amoxicillin, erythromycin, tetracycline, and neomycin (95–100%). *Staphylococcus* spp. had a high level of resistance to streptomycin, trimethoprim/sulfamethoxazole, tetracycline and gentamicin (80–100%). The MDR levels of *E. coli* and *Staphylococcus* spp. isolates from non-ruminants were 72.2 and 74.1%, respectively. Significantly higher resistance level were observed among isolates from non-ruminants compared to ruminants for tetracycline, amoxicillin, enrofloxacin, and trimethoprim/sulfamethoxazole.

## Introduction

The use of antimicrobial agents in animal farming is considered as one of the most essential factors that contribute to the emergence and dissemination of antibiotic resistant bacteria. Many of the antimicrobial agents such as ampicillin, gentamicin, and erythromycin used for livestock production and treatments are the same as, or closely related to, those used in medicines for humans. These drugs are categorized as “critically important antimicrobials” by the World Health Organization (WHO), and under the World Organization for Animal Health (OIE) Guidelines for antimicrobials, are categorized as “veterinary critically important antimicrobials” (1, 2), underscoring the significance of these agents for human as well as animal therapies.

In intensive food animal production, a substantial amount of antibiotics is used to prevent and treat various bacterial diseases. Unfortunately, a large quantity is also used as feed additives for the purpose of enhancing animal growth (3–5) and prophylaxis. The veterinary use of antimicrobial agents in food-producing animals for the purpose of growth promotion and disease prevention is therefore highlighted as one of the major risk factors or drivers for the emergence of antibiotic resistant-bacteria in animals and humans (6–8). Consequently, not only is the efficacy of antibiotics reduced, but also the risk of AMR pathogen transmission to humans is increased. In addition, the emergence of multi-drug resistant (MDR) bacteria poses an increasing challenge for veterinarians to render effective treatment to sick farm animals (9, 10). Bacteria such as *Escherichia coli* and *Klebsiella pneumoniae* are examples of two of most common bacteria that cause diseases in animals and are resistant to multiple antibiotics (11, 12). Other bacteria found in food animals such as *Salmonella, Campylobacter*, methicillin-resistant *Staphylococcus aureus* (MRSA), and *v*ancomycin-resistant *S. aureus* have adverse implications for public health (13–17). These bacteria can be transmitted to humans via the food chain and therefore may result in foodborne related illnesses that cannot be cured by commonly used antibiotics (18–20).

Antimicrobial resistance (AMR) surveillance has been established in Denmark and most developed countries in Europe to monitor the trends and patterns of antibiotic resistance in humans and food animals (21–23). In Malaysia, active and passive veterinary surveillance of AMR has recently begun as part of the National Action Plan for AMR (My-AP AMR 2017-2021) (24).

Currently, there are limited data on the extent and patterns of AMR in isolates from diseased food-producing animals. Laboratory diagnostic findings can be useful to provide an overview of the situation of AMR among diseased animals to support evidence-based decisions on the use of antibiotics. Hence, this study examined retrospective data from a collection of reports comprising clinical cases received by the Bacteriology Laboratory at the Faculty of Veterinary Medicine, Universiti Putra Malaysia, Serdang, Selangor, between 2015 and 2017. Information about the extent of resistance of bacteria from clinical cases will give a clearer picture of the seriousness of AMR in the country so that more appropriate antimicrobial agents can be selected for therapy and better guidelines for antibiotic use can be proposed.

## Materials and Methods

### Source of Data and Clinical Isolates

The data for this study originated from routine diagnostic cases received by the accredited Bacteriology Laboratory at the Faculty Veterinary Medicine of UPM Serdang, Selangor, Malaysia, from various veterinary health premises and animal facilities in Peninsular Malaysia. Cases from 1 January 2015 to 31 December 2017 were compiled and inputted into WHONET (vers 5.6, Boston, MA). The information included animal species, clinical history, specimen type (wounds/abscess, urine, lavage fluid, etc.), bacteria isolated, and antibiotic sensitivity testing (AST) results.

Standard microbiological procedures of isolation and identification to the species level were carried out using standard protocols (25). The antibiotic susceptibility test (AST) was performed using the Kirby Bauer's disc diffusion on Mueller Hinton Agar (MHA) method following the Clinical Laboratory Standards Institute (CLSI) guidelines (26). The AST results were grouped into three categories, viz. Resistant (R), Intermediate (I) and Susceptible (S) using established clinical breakpoints. For each bacterial species, the type of antibiotics tested differed according to the type of organism, the availability of antibiotics, and clinician's request.

Data from 241 cases were entered into Excel 2007 (Microsoft Office, 2017) and then transferred into WHONET 5.6 (27). A total of 701 isolates were obtained from various clinical samples of ruminants (cattle, goats and sheep) and non-ruminants (pigs and chicken) over the 3-year study period.

### Data Analysis

Data of isolates from ruminants and non-ruminants were analyzed separately in WHONET 5.6 (28, 29). The laboratory results from animal species (ruminants: cattle, goats and sheep; non-ruminants: chicken and pigs) were combined into two separate animal groups to increase data robustness as the number of isolates obtained for the different bacteria varied, and could be low in some bacteria. Therefore, data with fewer than 10 isolates were excluded from further analysis. Chi-square test was used to compare differences of AMR patterns between the two animal groups. The frequency of MDR to bacteria between ruminants and non-ruminants was also tabulated and compared. All the statistical analysis were performed using the SPSS (version 22.0, IBM, Armonk, NY: IBM Corp.) at significance level α=0.05.

## Results

### Descriptive Analysis of Clinical Samples

A total of 241 cases from diseased animals were received with requests for identification and AST. These cases comprised those of ruminants (*n* = 178, 73.9%), and non-ruminants (*n* = 63, 26.1%). From the cases, 395 specimens were received and a total of 701 isolates were obtained where more than half were from ruminants (*n* = 400, 57.1%), and the remaining from non-ruminants (*n* = 301, 42.9%). The specimens were from wounds/abscess (*n* = 112, 28.4%), post mortem (*n* = 109, 27.6%), milk (*n* = 87, 22.0%), feces (*n* = 55, 13.9%), and others, including nasal, ear and eye samples (*n* = 32, 8.1%).

**Figure 1:**
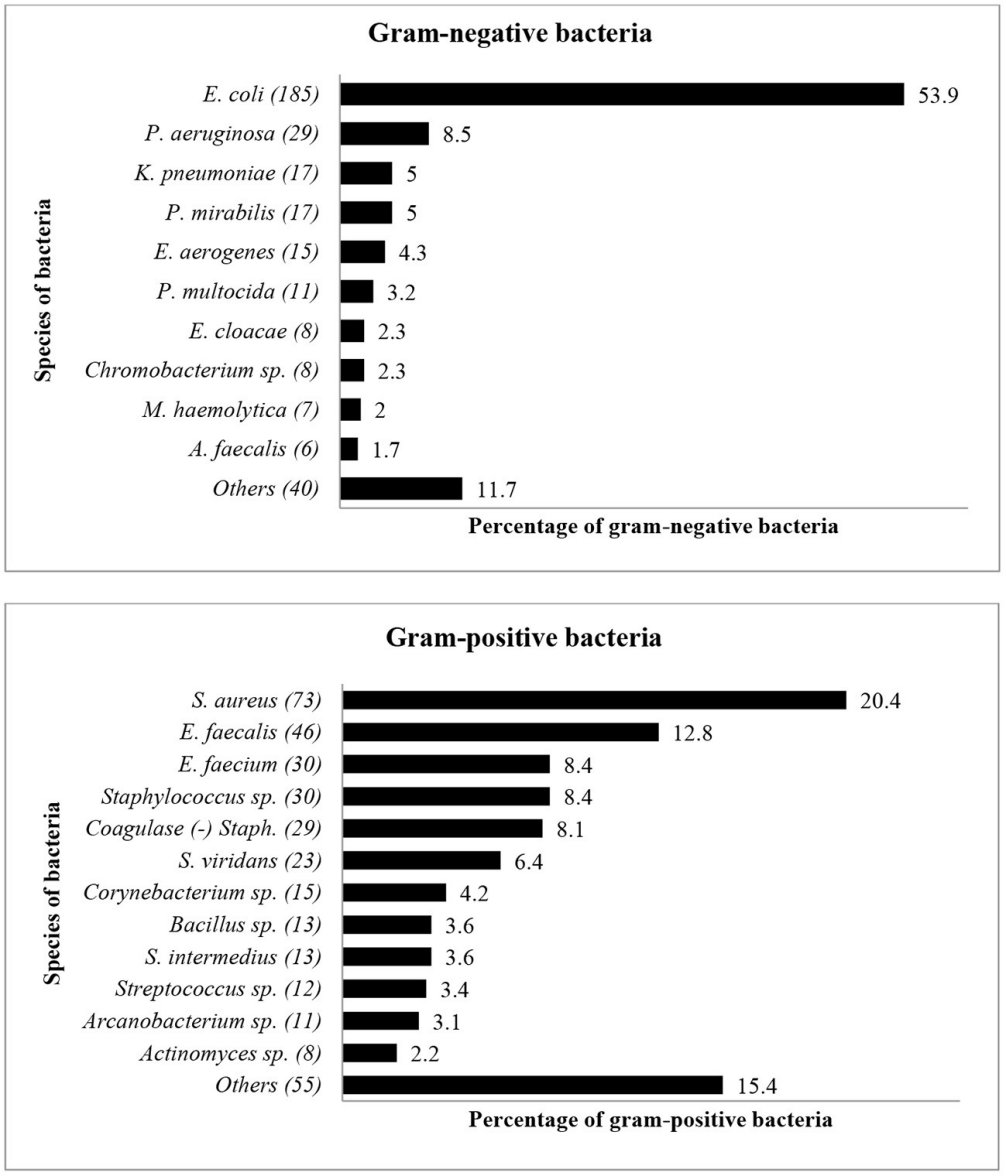
The distribution of bacterial species (Gram-negative and Gram-positive) from diseased livestock isolated between January 2015 and December 2017. Numbers inside brackets “()” indicate total number of bacterial isolates.

Fifty-nine bacterial species were identified during the study period, with *E. coli* (*n* = 185, 26%) and *S. aureus* (*n* = 73, 10%) being the predominant organisms isolated. Other bacteria included *Enterococcus faecalis* (*n* = 46, 7%), *Enterococcus faecium* (*n* = 30, 4%), *Pseudomonas aeruginosa* (*n* = 29, 4%) and coagulase-negative *Staphylococcus* spp. (*n* = 29, 4%). Figure 1 shows the distribution and percentages of species of bacteria isolated between 2015 and 2017. *Escherichia coli* (*n* = 185, 26%) was the most common gram-negative bacteria isolate, while *S. aureus* (*n* = 73, 10%) was the most common gram-positive bacteria isolated from livestock.

### Antimicrobial Resistance of Clinical Isolates From Ruminants

Between 2015 and 2017, 178 cases of diseased ruminants (cattle *n* = 93, goats *n* = 78, sheep *n* = 7) were received for isolation and identification of bacteria and AST. Four hundred isolates were recovered from 235 clinical specimens. Of these, one third (37%, 87/235) were from milk, followed by wounds/abscess (33.2%, 78/235), post-mortem (17.4%, 41/235), feces (8.5%, 20/235), and others including nasal, ear, and eye samples (3.8%, 9/235). Overall, *E. coli* (19.3%, 77/400), *S. aureus* (18.3%, 73/400) and coagulase (negative) *Staphylococcus* spp. (5.8%, 23/400) were most frequently isolated (Table 1).

**Table 1:** Species of bacteria isolated from samples of diseased ruminants received by the Bacteriology Laboratory between 2015 and 2017.

**Clinical samples**	***N***	**(%) *Escherichia coli* (*n* = 77)**	**(%) *Staphylococcus aureus* (*n* = 73)**	**(%) *Coagulase* (-)*Staphylococcus* (*n* = 23)**	**(%) Others (*n* = 227)**
Post mortem	41	20 (26%)	12 (16.4%)	0	41 (18.1%)
Wounds/abscess	78	26 (33.8%)	26 (35.6%)	1 (1.3%)	78 (34.4%)
Milk	87	8 (10.4%)	28 (38.4%)	22 (25.3%)	79 (34.8%)
Feces	20	20 (26%)	4 (5.5%)	0	20 (8.8%)
Others	9	3 (3.9%)	3 (4.1%)	0	9 (4%)
Total	235	77 (100%)	73 (100%)	23 (100%)	227 (100%)

*N = Total number of clinical samples; n = Total number of bacterial isolates*.

About 72% (287/400) of bacteria isolated had multi-drug resistance (MDR) i.e., resistant to at least three or more antibiotic classes; 25% (100/400) were resistant to one or two antibiotic classes and only 3% (13/400) were susceptible to all tested antibiotics.

### Antibiogram Based on Species of Bacteria Isolated From Ruminants

**Figure 2:**
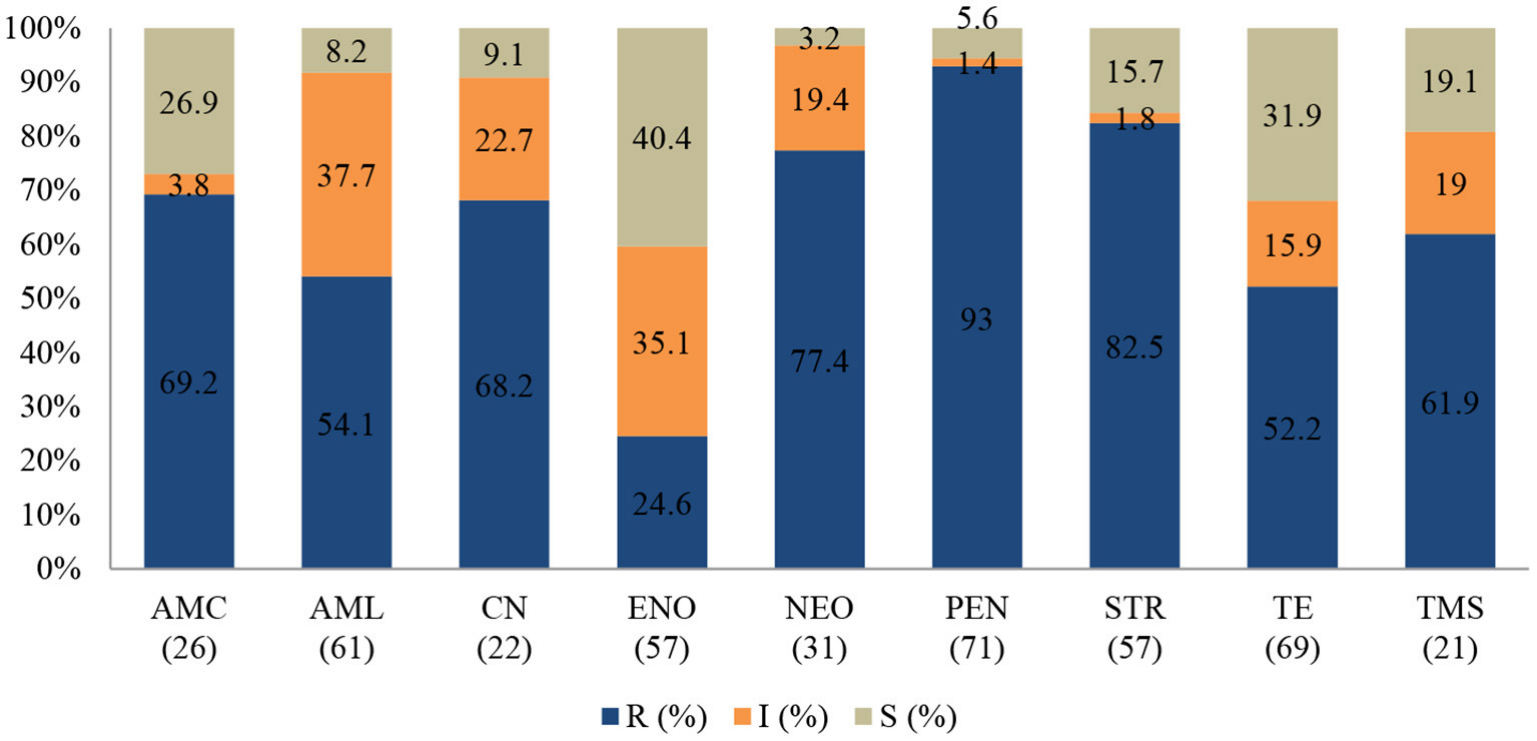
Antibiotic susceptibility pattern of *Escherichia coli* isolates from diseased ruminants (January 2015-December 2017). R-resistance; I-intermediate; S-susceptible; AMC-amoxicillin/clavulanic acid; AML-amoxicillin; CN-gentamicin; ENO enrofloxacin; NEO-neomycin; PEN-penicillin; STR-streptomycin; TE-tetracycline; TMS-trimethoprim/sulfamethoxazole; Numbers inside brackets “()” indicate total number of tested isolates for each antibiotic.

**Figure 3:**
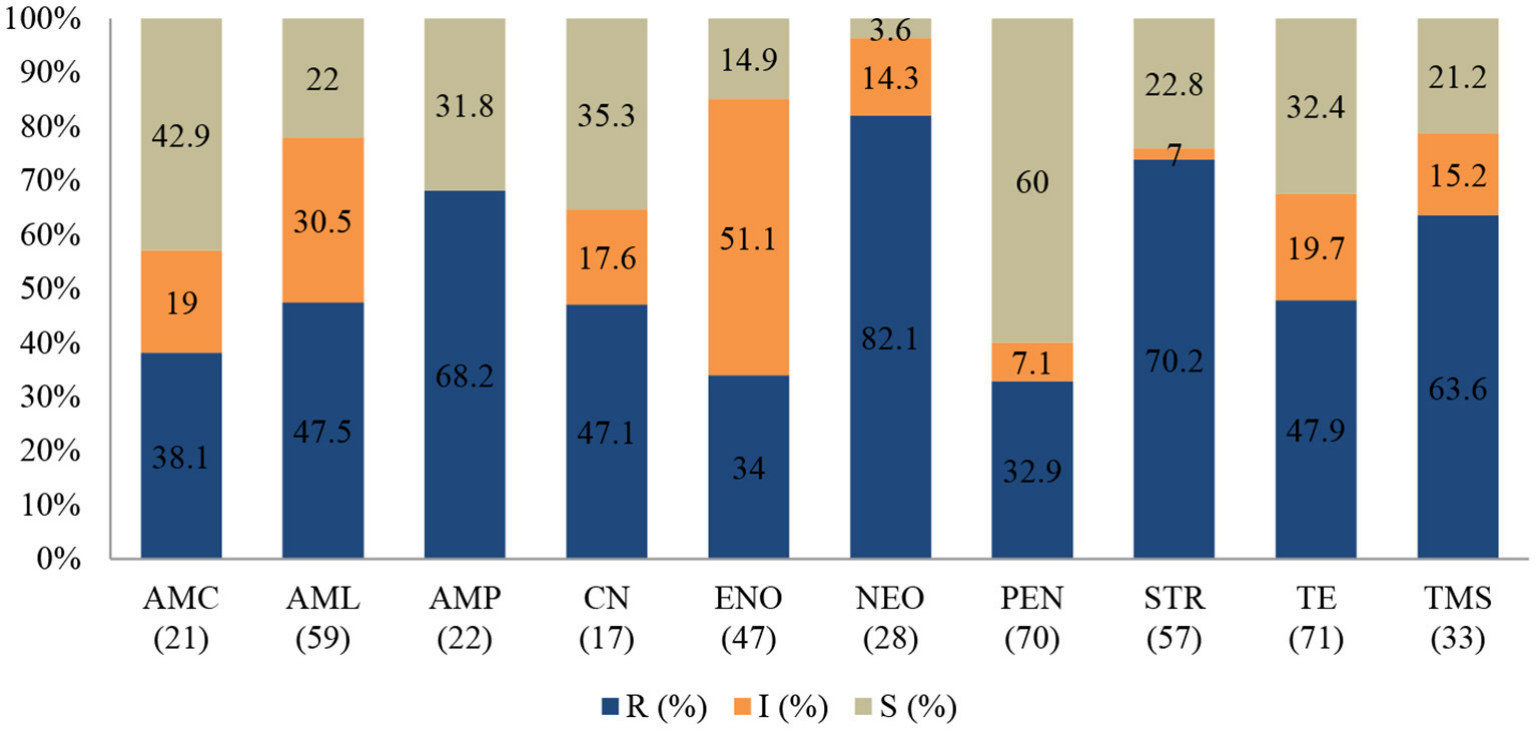
Antibiotic susceptibility pattern of *Staphylococcus aureus* isolates from diseased ruminants (January 2015-December 2017) R-resistance; I-intermediate; S-susceptible; AMC-amoxicillin/clavulanic acid; AML-amoxicillin; AMP-ampicillin; CN-gentamicin; ENO-enrofloxacin; NEO-neomycin; PEN-penicillin; STR-streptomycin; TE-tetracycline; TMS-trimethoprim/sulfamethoxazole; Numbers inside brackets “()” indicate total number of tested isolates for each antibiotic.

**Figure 4:**
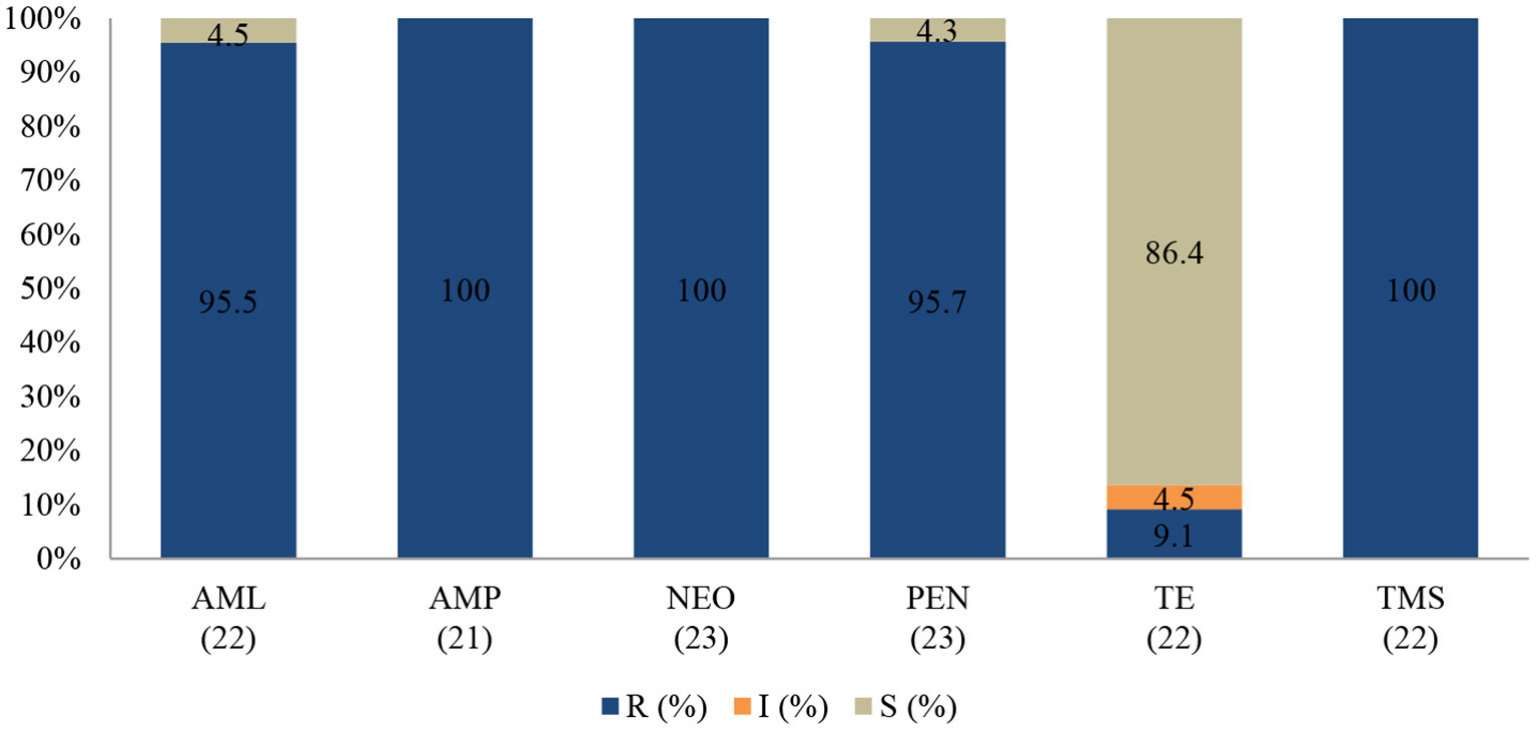
Antibiotic susceptibility pattern of Coagulase-negative *Staphylococcus* spp. isolates from diseased ruminants (January 2015-December 2017). R-resistance; I-intermediate; S-susceptible; AML-amoxicillin; AMP-ampicillin; NEO-neomycin; PEN-penicillin; TE-tetracycline; TMS-trimethoprim/sulfamethoxazole; Numbers inside brackets “()” indicate total number of tested isolates for each antibiotic.

**Figure 5:**
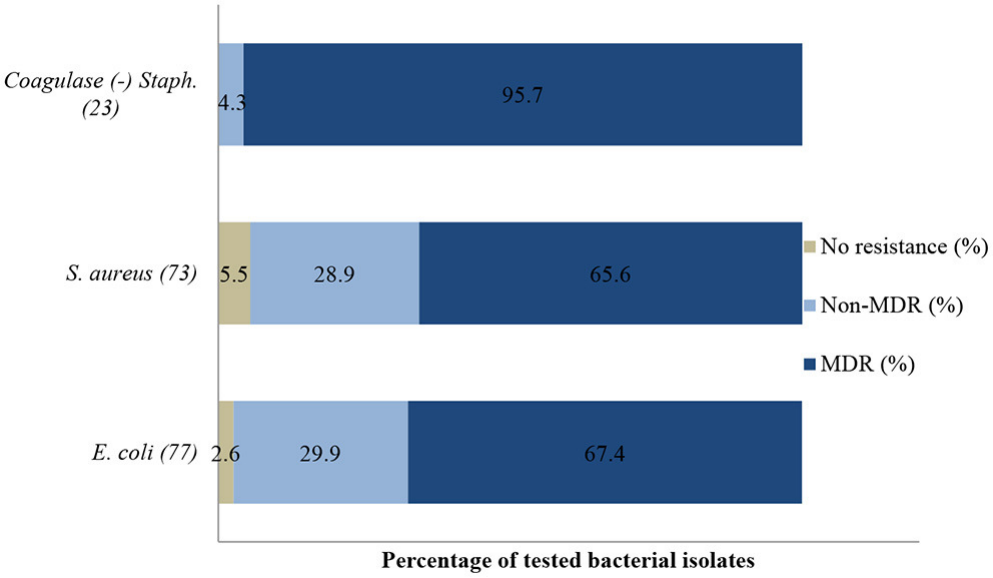
Multi-drug resistance of clinically important bacterial pathogens from diseased ruminants between 2015 and 2017. Numbers inside brackets “()” indicate number of isolates detected; those on bars indicate percentage per organism; Non-MDR–Only resistant to 1 or 2 classes; MDR–multi-drug resistant.

An antibiogram of the bacterial species isolated is presented in Figures 2–**4**. Most of the selected bacteria were tested against fluoroquinolone, penicillin, tetracycline and aminoglycosides. The MDR level for the selected bacteria for 2015–2017 is illustrated in **Figure 5**.

#### Escherichia coli

Figure 2 shows that the highest level of resistance was to penicillin (93%; 95% CI = 83.7–97.4) followed by streptomycin (82.5%; 95% CI = 69.7–90.9), and neomycin (77.4%; 95% CI = 58.4–89.7. A statistically significant (*p* = 0.004) decline of the resistance level to tetracycline was observed, with 90% (95% CI = 68.3–98.8) in 2015 to 46.4% (95% CI = 27.5–66.1) in 2017.

Similarly, a significant (*p* = 0.001) decrease of resistance level (93.8%; 95% CI = 69.8–99.8) to (25%; 95% CI = 3.2–65.1) to amoxicillin/clavulanic acid was also recorded between 2015 and 2017. Multi-drug resistance (MDR) in **Figure 5** shows that more than 67% (52/77) of *E. coli* isolated were resistant to multiple classes of antibiotics; 29.9% (23/77) were resistant to one or two antibiotic classes and only 2.6% (2/77) were susceptible to all antibiotics tested. In addition, more than 90% of the isolates were not susceptible (intermediate or resistant) to four of nine tested antibiotics.

#### Staphylococcus aureus

*Staphylococcus aureus* was the second most common pathogen isolated from ruminants. The proportion of AMR (Figure 3) among the 73 *S. aureus* isolates resistant to neomycin was 82.1% (95% CI = 62.4–93.2), 70.2% to streptomycin (95% = 56.5–81.2), and 68.2% to ampicillin (95% CI = 45.1–85.3). However, the resistance levels to penicillin (32.9%; 95% CI = 22.4–45.3), and amoxicillin/clavulanic acid (38.1%; 95% CI = 19.0–61.3) in *S. aureus* were relatively lower.

A statistically significant increase in resistance proportion among *S. aureus* to neomycin (*p* = 0.021) from 94.7% (95% CI = 74–99.9) in 2015 to 100% (95% CI = 19.4–99.4) in 2017 was observed, to ampicillin (*p* = 0.001) from 14.3% (95% CI = 0.8–58.0) in 2015 to 93.3% (95% CI = 66.0–99.6) in 2017, and to gentamicin (*p* = 0.002), from 40% (95% CI = 0.3–44.5) in 2015 to 100% (95% CI = 56.1–100) in 2017 were observed. Generally, 65.6% (48/73) of *S. aureus* isolated were MDR; 28.9% (21/73) were resistant to one or two antimicrobial agents tested and 5.5% (4/73) were not susceptible to all antibiotics tested (**Figure 5**). More than 90% of the isolates were not susceptible to one of ten tested antibiotics.

### Coagulase-Negative *Staphylococcus* spp.

None of the tested isolates of coagulase (negative) *Staphylococcus* were susceptible to the action of neomycin (100%; 95% CI = 82.2–100), ampicillin (100%; 95% CI = 80.8–100), and trimethoprim-sulfamethoxazole (100%; 95% CI = 81.5–100). The resistance rates were also high for penicillin (95.7%; 95% CI = 76.1–99.8) and amoxicillin (95.5%; 95% CI = 75.2–99.8). Interestingly, more than 80% of isolates remained sensitive to tetracycline (Figure 4).

No statistically significant resistance trends to all tested antibiotics were observed over the study period (*P* > 0.05). The MDR of coagulase (negative) *Staphylococcus* (Figure 5) over the three-year period was 95.7% (22/23), with 4.3% (1/23) being resistant to one or two antimicrobial agents tested. More than 90% of the isolates were no longer susceptible to five of six tested antibiotics.

### Antimicrobial Resistance of Clinical Isolates From Non-ruminants

Over the 3-year study period, 63 cases from non-ruminants (pigs *n* = 34 and chicken *n* = 29,) were received for identification and AST. A total of 160 clinical specimens were from post-mortem (42.5%, 68/160), followed by wounds/abscess and feces which accounted for (21.25%, 34/160) and (21.9%, 35/160), and other samples such as nasal and eye swabs (14.4%, 23/160).

From these specimens, a total of 301 isolates were recovered. *E. coli* was the most frequent isolate (35.9%, 108/301), followed by *Staphylococcus* spp. (9%, 27/301) and *E. faecalis* (9%, 27/301) (Table 2). More than 82.1% (247/301) of bacteria isolated were MDR, i.e., resistant to at least three or more antibiotic classes; 17.6% (53/301) were resistant to one or two antibiotic classes and 0.3% (1/301) of the isolates were susceptible to all tested antibiotics.

**Table 2:** Species of bacteria isolated from samples from diseased non-ruminants received by the Bacteriology Laboratory between 2015 and 2017.

**Clinical samples**	***N***	**(%) *Escherichia coli* (*n* = 108)**	**(%) *Staphylococcus* sp. (*n* = 27)**	**(%) *Enterococcus faecalis* (*n* = 27)**	**(%) Others (*n* = 139)**
Post mortem	68	58 (53.7%)	13 (48%)	20 (74%)	60 (43.2%)
Wounds/abscess	34	21 (19.4%)	14 (52%)	2 (7.4%)	34 (24.5%)
Feces	35	28 (26%)	0	5 (18.5)	24 (17.3%)
Others	23	1 (0.9%)	0	0	21 (15%)
Total	160	108 (100%)	27 (100%)	27 (100%)	139 (100%)

*N = Total number of clinical samples; n = Total number of bacterial isolates*.

### Antibiogram Based on Species of Bacteria Isolated From Non-ruminants

**Figure 6:**
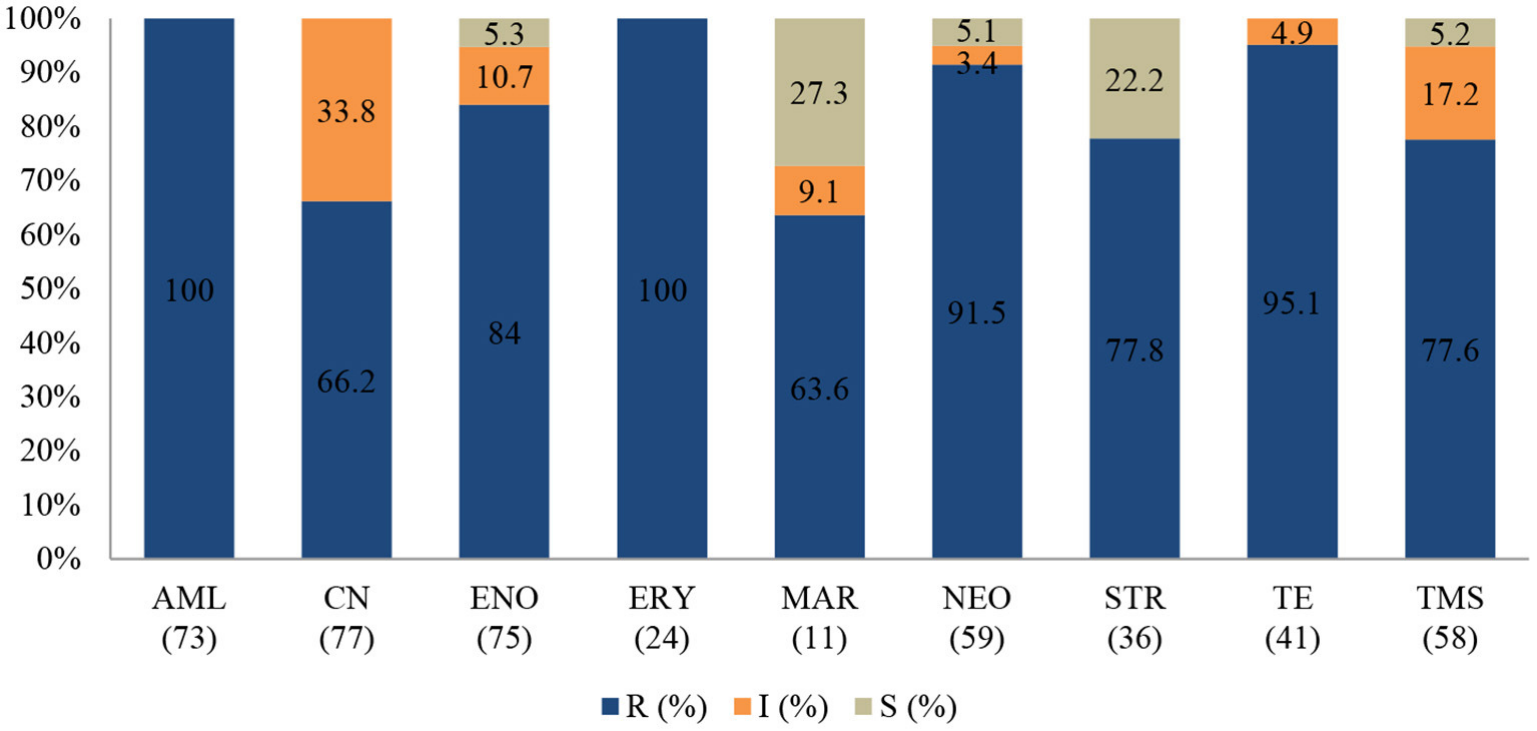
Antibiotic susceptibility pattern of *Escherichia coli* isolates from diseased non-ruminants (January 2015-December 2017). R-resistance; I-intermediate; S-susceptible; AML-amoxicillin; CN-gentamicin; ENO-enrofloxacin; ERY-erythromycin; MAR-marbofloxacin; NEO-neomycin; STR-streptomycin; TE-tetracycline; TMS-trimethoprim/sulfamethoxazole; Numbers inside brackets ‘()’ indicate total number of tested isolates for each antibiotic.

**Figure 7:**
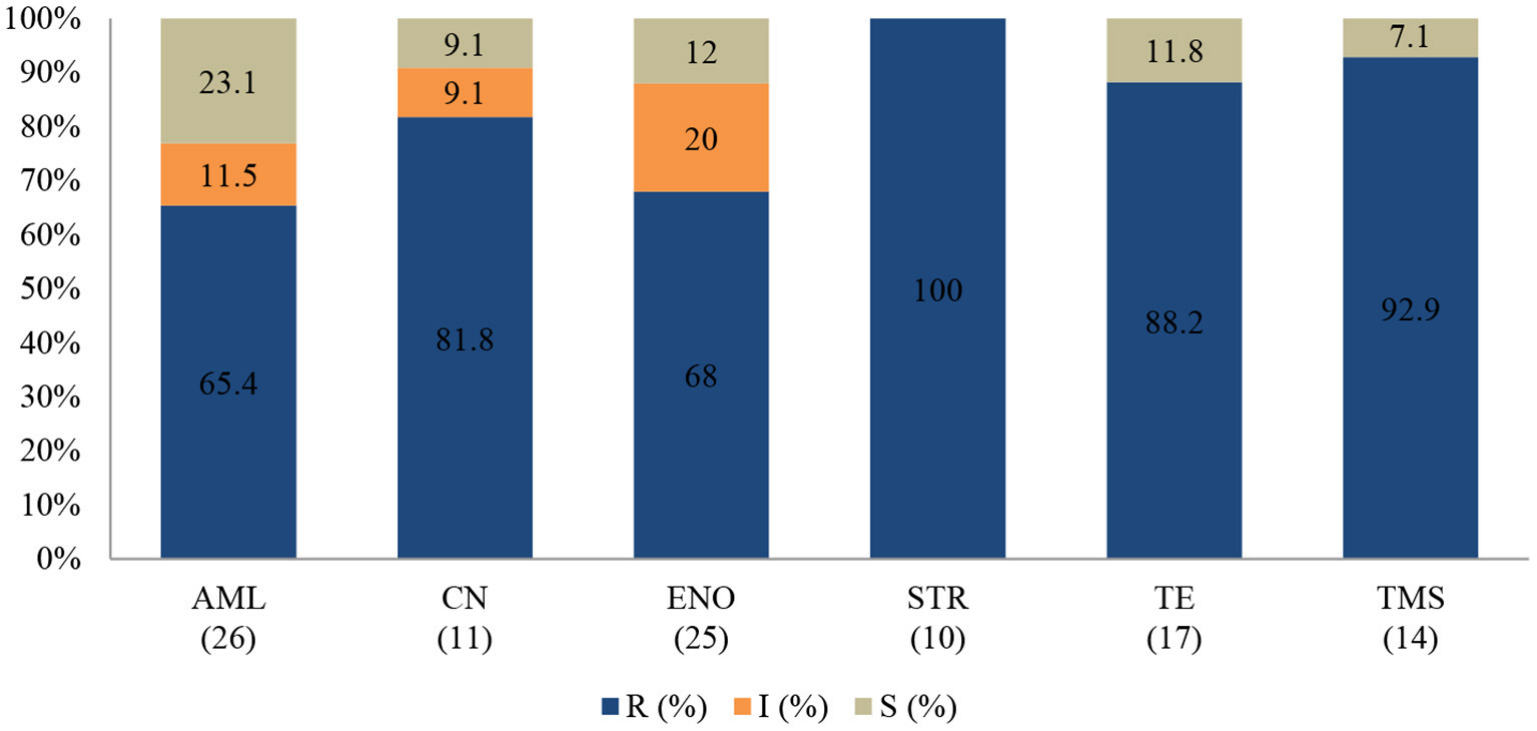
Antibiotic susceptibility pattern of *Staphylococcus* spp. isolates from diseased non-ruminants (January 2015-December 2017). R-resistance; I-intermediate; S-susceptible; AML-amoxicillin; CN-gentamicin; ENO-enrofloxacin; STR-streptomycin; TE-tetracycline; TMS-trimethoprim/sulfamethoxazole; Numbers inside brackets “()” indicate total number of tested isolates for each antibiotic.

**Figure 8:**
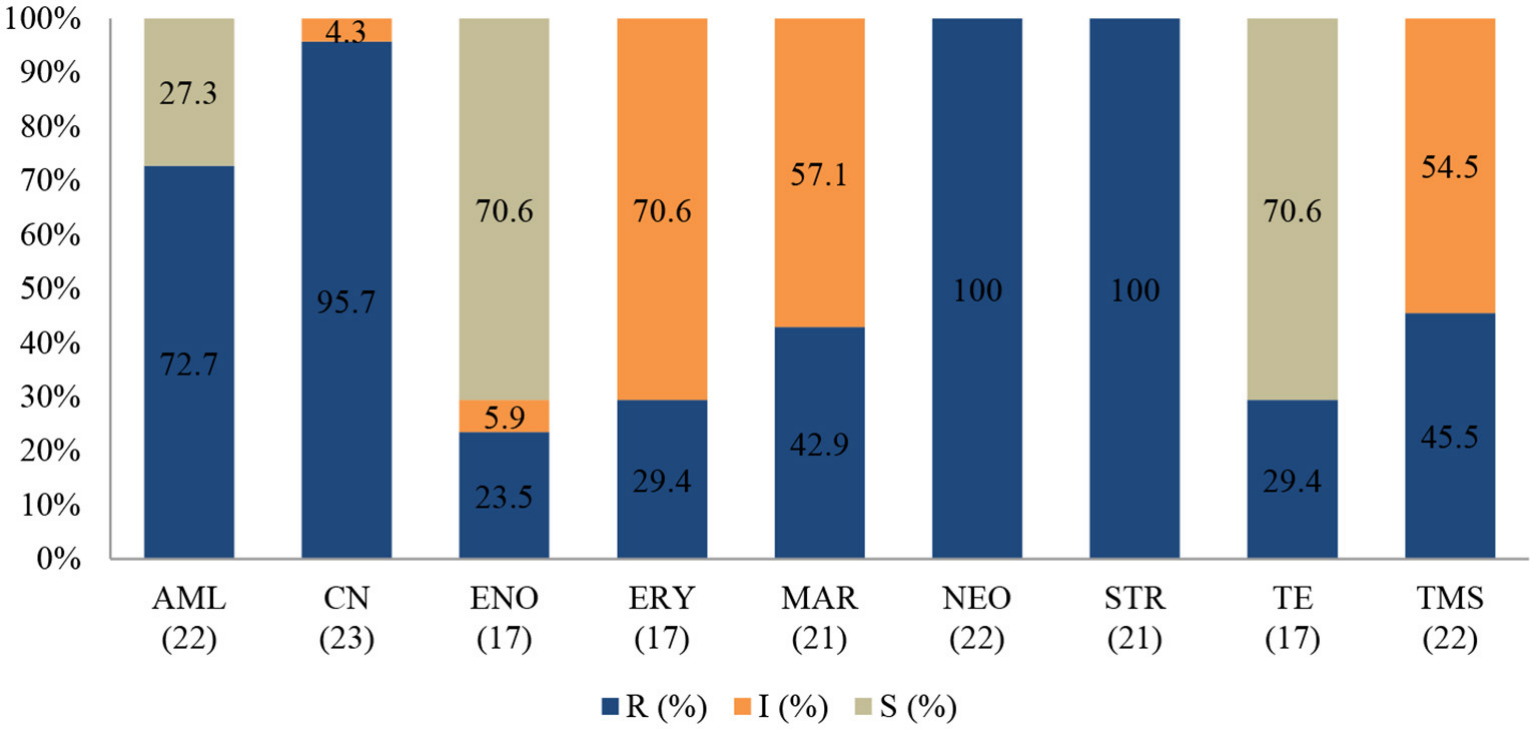
Antibiotic susceptibility pattern of *Enterococcus faecalis* isolates from diseased non-ruminants (January 2015-December 2017). R-resistance; I-intermediate; S-susceptible; AML-amoxicillin; CN-gentamicin; ENO-enrofloxacin; ERY-erythromycin; MAR-marbofloxacin; NEO-neomycin; STR-streptomycin; TE-tetracycline; TMS-trimethoprim/sulfamethoxazole; Numbers inside brackets “()” indicate total number of tested isolates for each antibiotic.

**Figure 9:**
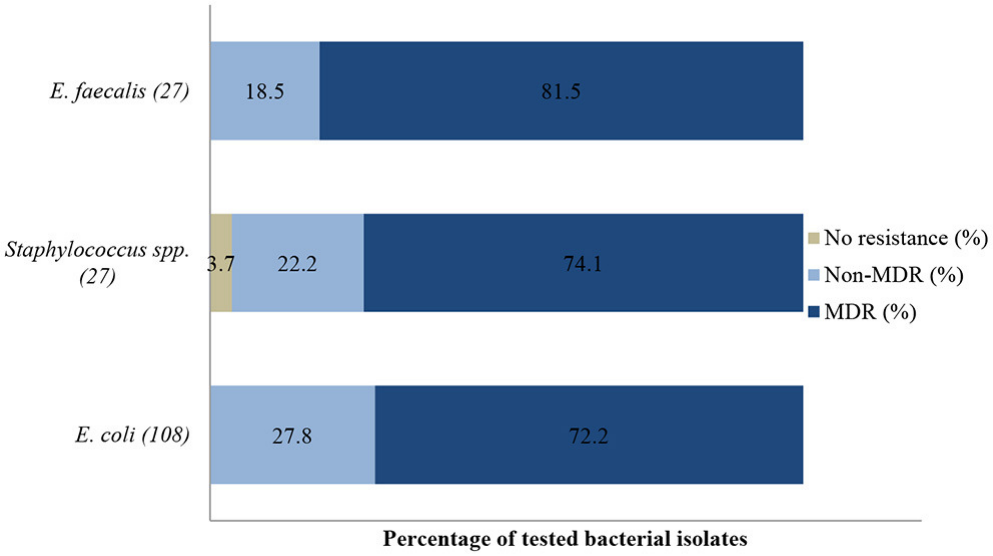
Multidrug resistance of clinically important bacterial pathogens from diseased non-ruminants between 2015 and 2017. Numbers inside brackets “()” indicate number of isolates detected; those on bars indicate percentage per organism; MDR–multi-drug resistant. Non-MDR-Only resistant to 1 or 2 classes.

An antibiogram of the bacteria species isolated is presented in Figures 6–**8**. The MDR levels for the selected bacteria during the study period are illustrated in **Figure 9**.

#### Escherichia coli

In the antibiogram of *E. coli* presented in Figure 6, complete or almost complete resistance was observed against amoxicillin (100%; 95% CI = 93.8–100), erythromycin (100%; 95% CI = 82.8–100), tetracycline (95.1%; 95% CI = 82.2–99.1) and neomycin (91.5%; 95% CI = 80.6–96.8). Chi-square analysis determined that no significant differences were observed in the proportions of resistant isolates between time periods for all antibiotics tested. Generally, 72.2% (78/108) of *E. coli* isolates were MDR while 27.8% (30/108) were resistant to one or two antimicrobial agents *tested (****Figure 9****). More than 90% of isolates were not-susceptible to seven of nine tested* antibiotics.

#### *Staphylococcus* spp.

All *Staphylococcus* spp. isolates were resistant to streptomycin (100%; 95% CI = 65.5–100). The resistance (Figure 7) was also high for trimethoprim/sulfamethoxazole (92.9%; 95% CI = 64.2–99.6), tetracycline (88.2%; 95% CI = 62.2–97.9), and gentamicin (81.8%; 95% CI = 47.7–96.8). A significant increase in resistance level was seen for amoxicillin (*P* = 0.003), from 50% (95% CI = 2.7–97.3) in 2015 to 100% (95% CI = 75.3–100) in 2017. The MDR of *Staphylococcus* spp. over the 3-year period was 74.1% (20/27), while 22.2% (6/27) were resistant to one or two classes, and 3.7% (1/27) of the tested *Staphylococcus* spp. isolates were susceptible to all classes of antimicrobial agents (**Figure 9**). More than 90% of the isolates were not susceptible to three of six tested antibiotics.

#### Enterococcus faecalis

The *E. faecalis* antibiotic resistance level between 2015 and 2017 is presented in Figure 8. Resistance was 100% to streptomycin (95% CI = 80.8–100), 100% to neomycin (95% CI = 81.5–100), 95.7% to gentamicin (95% CI = 76.1–99.8), and 72.7% to amoxicillin (95% CI = 49.5–88.4). The resistance levels to enrofloxacin (23.5%; 95% CI =7.8–50.2) and tetracycline (29.4%; 95% CI 11.4–55.9) were lower. For tetracycline and erythromycin, no statistically significant resistance trends were observed over the years. On the other hand, a significant decrease (*P* = 0.00) in resistance levels against enrofloxacin from 100% (95% CI = 39.6–100) in 2015 to 0% (95% CI = 0.0–30.1) in 2017 was recorded. The MDR was more than 81.5% (22/27) and only 18.5% (5/27) were resistant to one or two antimicrobial agents tested (Figure 9). More than 90% of *E. faecalis* isolates were not susceptible to six of nine antibiotics.

### Differences Between Resistance Profile of Isolates of Ruminants and Non-ruminants

Antimicrobial resistance profiles among *E. coli* isolates are presented in Table 3. In both ruminants and non-ruminants, *E. coli* was frequently resistant to streptomycin and neomycin (77 to 92%) as well as trimethoprim/sulfamethoxazole (62 to 78%). Generally, *E. coli* isolates from non-ruminant showed higher levels of resistance toward all antimicrobial agents tested, with 100% resistance to amoxicillin and erythromycin; they were also highly resistant toward enrofloxacin. In contrast, *E. coli* isolates from ruminants showed high resistance toward penicillin (93%).

**Table 3:** Antimicrobial resistance profiles among *E. coli* isolates from diseased ruminants and non-ruminants between 2015 and 2017.

	**Resistance % (95% CI)**			
	**Ruminants**		**Non-ruminants**	
**Antimicrobial agents**	***N***	***Escherichia coli***	***N***	***Escherichia coli***
Penicillin	71	93 (83.7–97.4)	NA	NA
Tetracycline	69	52.2 (39.9–64.2)	41	95.1 (82.2–99.1)^*^
Amoxicillin	61	54.1 (40.9–66.7)	73	100 (93.8–100)^*^
Streptomycin	57	82.5 (69.7–90.9)	36	77.8 (60.4–89.3)
Enrofloxacin	57	24.6 (14.6–38.1)	75	84 (73.3–91.1)^*^
Neomycin	31	77.4 (58.4–89.7)	59	91.5 (80.6–96.8)
Amoxicillin/clavulanic acid	26	69.2 (48.1–84.9)	NA	NA
Trimethoprim/sulfamethoxazole	21	61.9 (38.7–81.0)	58	77.6 (64.4–87.1)
Gentamicin	22	68.2% (45.1–85.3)	77	66.2 (54.4–76.3)
Erythromycin	NA	NA	24	100 (82.8–100)
Marbofloxacin	NA	NA	11	63.6 (31.6–87.6)

*NA, Insufficient data (fewer than 10 isolates) available; N, Total number of tested isolates for each antibiotic*.

* *Significant difference between ruminants and non-ruminants, P < 0.05*.

Analysis of the patterns of resistance was limited to antimicrobial agent groups with *n* > 10 isolates; others with <10 isolates were excluded from the analysis. Statistically significant differences between the frequency of resistance of ruminants and non-ruminants were observed between *E. coli* resistance levels to tetracycline, enrofloxacin, and amoxicillin.

Multi-drug resistance level during the study period (2015–2017) showed that frequency of MDR *E. coli* isolates decreased from 88.6% in 2015 to 53.7% in 2017. However, the difference was not significant (67.4 vs. 72.2%; χ^2^= 0.473, *p* = 0.492) in both animal groups.

## Discussion

According to the list of antibiotics under WHO and OIE (1, 30), several classes of antibiotics overlapped among those listed under the critically important antibiotics and veterinary critically important antibiotics (VCIA), including 3rd and 4th generation cephalosporin, macrolide, fluoroquinolone, aminoglycosides, and penicillin. In addition, those listed under VCIA include tetracycline, sulphonamide, and penicillin. Therefore, analysis on veterinary data from clinical isolates is an important component of a strategic action plan for AMR as it provides background AMR information and complements active surveillance data, thus reflecting the overall antimicrobial resistance situation in a country. Unfortunately, as there are few reports published on AMR trends among isolates from diseased animals, it is challenging to compare our findings as most publications are based on samples from healthy animals or animal food products.

*Escherichia coli, Staphylococcus aureus* and coagulase (negative) *Staphylococcus* spp. are the most frequently isolated agents from diseased ruminants. *Escherichia coli* isolates are frequently highly resistant or not susceptible to most antibiotics categorized as VCIA tested, such as penicillin, streptomycin, neomycin and gentamicin. This is contrary to the report from a veterinary regional laboratory in the northern part of Peninsular Malaysia that recorded lower levels of resistance for streptomycin (26%), neomycin (33%), and gentamycin (23%) from diseased large and small ruminants (31). About 60% of *E. coli* isolates in this study were not susceptible to enrofloxacin, which is consistent to that reported in diseased small ruminants in India (32) (65.5%), but higher than that reported in clinical isolates from cows (16.4%) in China (33). The same report from India indicated *E. coli* was mainly sensitive to gentamicin, in contrast to the study in China, and to our study that showed clinical *E. coli* isolates to be more than 85% resistant to gentamicin. In fact, 90% of all *E. coli* isolates from diseased ruminants in this study were no longer susceptible to four of nine tested antibiotics. The level of MDR (67%) was higher than that reported for *E. coli* in the same group of animals (31) and in isolates from cows in China (33).

*Staphylococcus aureus* demonstrated very high resistance to most antibiotics tested, such as neomycin, streptomycin, ampicillin and trimethoprim/sulfamethoxazole, and increasing non-susceptibility against enrofloxacin and amoxicillin. Coagulase (negative) *Staphylococcus* was completely resistant to most tested antibiotics except for tetracycline. Contrary to this finding, lower levels of resistance against the same aforementioned antibiotics were reported in Algeria (34) and in Turkey (35). These researchers reported resistance levels of *S. aureus* and coagulase (negative) *Staphylococcus* isolates from cows with mastitis to trimethoprim/sulfamethoxazole (ranging from 2 to 45%) and neomycin (ranging from 0 to 30%). Phophi et al. (36) reported about 90% resistance of coagulase negative *Staphylococcus* to ampicillin and penicillin and 51% MDR, both figures being similar to our observation in this study. The lowest resistance was demonstrated in *S. aureus* isolates against penicillin (32.9%), which was lower than that previously reported against the same antibiotic (44–54%) in raw milk in Egypt (37). Similarly, sensitivity level was reported in mastitic milk samples in Switzerland at 10.6, 8.2, and 7.7% against erythromycin, penicillin and tetracycline, respectively (38). *S. aureus* and coagulase (negative) *Staphyloccoccus* were both more than 90% resistant to neomycin. This could be attributed to the wide use of the aminoglycosides for the treatment of mastitis (39). A significant increasing resistance trend was also reported for *S. aureus* recovered from dairy cattle with suspected mastitis in the Animal Health Diagnostic Laboratory of Michigan in the United States (40) between 1994 and 2000. The researchers reported that *S. aureus* resistance trends to gentamicin increased from 95.4% in 1994 to 100% in 2000.

Given that ruminants in Malaysia are mainly raised in extensive or semi-intensive systems where antibiotics are less widely administered (41), it is worrying to observe that more than 60% of the most common isolated pathogens from diseased animals are MDR. This could probably be due to the intermittent use of various types of antibiotics for therapy and the possible transfer of resistant-bacteria from the environment. Ruminants generally have a closer interaction with organisms in soil and water due to feeding and grazing activities, and as such, are frequently exposed to resistant bacteria in the environment that serves as a reservoir for antibiotic resistance (42, 43). In our previous study, we have reported that *Salmonella* from free-range chickens which were rarely given antibiotics acquired resistance to colistin and were MDR (44).

Generally, bacteria from diseased non-ruminants in this study demonstrated higher resistance levels against the majority of the tested antimicrobials, including fluoroquinolone, compared to ruminants. In the former, *E. coli* isolates displayed complete resistance levels against amoxicillin and erythromycin, as well as more than 90% resistance levels against tetracycline and neomycin. These findings are consistent with the trend for tetracycline as reported by Shahaza et al. (31) and corroborate with other similar studies, where *E. coli* has been reported as having high resistance levels against the aforementioned antimicrobials in Serbia (45) and also elsewhere around the world (46, 47). In the study by Dosen et al. (45), the authors reported that high levels of *E. coli* isolates recovered from necropsied pigs were not susceptible to tetracycline (93.55%), amoxicillin (73.34%), and neomycin (61.3%). Furthermore, researchers from Ireland also reported high levels of resistance of *E. coli* isolates against tetracycline (100%) and streptomycin (97.3%) in samples recovered from routine diagnostic cases at the University Veterinary Hospital, Ireland (48). *E. coli* isolates from this study demonstrated high levels of non-susceptibility against fluoroquinolones enrofloxacin (94.7%) and marbofloxacin (72.7%). Researchers have shown that resistance to fluoroquinolones was often associated with tetracycline and trimethoprim/sulfamethoxazole resistance (49), consistent with high levels of tetracycline (95.5%) and trimethoprim/sulfamethoxazole (77.6%) resistance observed in this study. High resistance levels by *E. coli* isolates against 3rd generation fluoroquinolones such as the aforementioned could be attributed to a few factors. Fluoroquinolone is a broad-spectrum antimicrobial and is frequently used in food-producing animal settings (50, 51) as a blanket prevention or treatment of infections such as enteric and respiratory infections (52). For Gram-positive *Staphylococcus* spp. and *E. faecalis*, complete resistance was demonstrated against streptomycin (both 100%) as well as high resistance levels against gentamicin (81.8%; 95.7%). In the study of Liu et al. (53), the highest level of *Staphylococcus* spp. resistance manifested was against gentamicin (85%). Contrary to our findings, study of *E. faecalis* isolates recovered from fecal samples of broiler breeders in Korea (54) demonstrated low resistance levels against gentamicin (10.5%) and streptomycin (16.2%).

*Escherichia coli* is regarded as an excellent sentinel for AMR in a wide range of animal species (55, 56). Hence, it is a suitable candidate for comparing resistance profiles between animal groups. In addition, most antibiotic classes used to treat *E. coli* infection are shared between animal species. *E. coli* was the most commonly isolated pathogen in this study, consistent with other reports (31, 57). Statistically significant differences were observed for the antimicrobial resistance pattern between the two groups. *E. coli* isolates from non-ruminants demonstrated higher resistance levels compared to the isolates from ruminants for tetracycline, amoxicillin and enrofloxacin. Other researchers in Tunisia (58), UK (59), and China (60, 61) reported similar resistance trends in non- ruminants against tetracycline (74.7–96.7%), amoxicillin (57–86.7%), and enrofloxacin (64.5–78.9%). Studies by Abbassi et al. (56) and Lei et al. (60) also corroborate with our findings, where *E. coli* isolates from ruminants generally demonstrated lower resistance trends than those from poultry and swine. There are many possible reasons for the observed differences in the resistance pattern between the isolates from ruminants and non-ruminants. In the Malaysian setting, we believe that a major reason is the prolonged exposure and lengthy use of antimicrobials in the intensive production systems of chicken and pigs compared to extensive or semi-intensive production settings (where antimicrobials are used less widely) for cattle and goats (62, 63). For example, heavy usage of antimicrobials in chicken and pig industries has been documented in various reports (64) and linked to the observed increasing resistance levels of bacterial isolates from these animal species. Significantly larger quantities of antibiotics are used to produce the same volume of meat from pigs than from ruminants. For instance, it has been reported that 45 mg of antibiotics are needed to produce 1 kg of beef, while 172 mg are needed to produce the same weight of pork (65–67).

High levels of MDR of clinically important bacterial pathogens isolated from food animals have serious implications. In this study, we found an alarming frequency of multidrug resistant and extensively drug resistant clinical isolates in both animal groups. MDR of clinically important bacterial pathogens reduces the therapeutic options for disease incidence. This not only impacts food security but also increases the chances of transmission and dissemination of resistant pathogens to other bacteria by horizontal transfer of resistance genes to the same or different species of bacteria (68, 69). Consequently, resistant organisms can directly or indirectly be transferred to humans via food or the environment. Recently, MDR has been widely reported especially in commensal *E. coli* which has the versatility to develop and choose several mechanisms to fight off the effect of antimicrobial agents (70) and donate resistance materials to other bacteria (71).

There are inherent limitations to our findings. Sampling bias represents an important limitation of this study. This is because samples were received from diseased animals which might or might not have been recently or previously treated with antibiotics. Besides, the history of antibiotic usage at the farm was not known to us. Thus, the data from this study do not necessarily represent livestock in Peninsular Malaysia and may not fully reflect the overall picture of AMR pattern and trends from diseased food animals. Another bias is that our findings could not differentiate whether the isolated bacteria were part of normal flora or were causative agents of the infection. Furthermore, the antimicrobial agents were not tested in a consistent manner over the study period because antimicrobial agents were applied depending on the type of bacterial pathogens, clinician's opinion, clients' request and the availability of the antimicrobial agents in the diagnostics laboratory.

## Conclusion

This study provides an overall picture of the resistance trends in clinically important *E. coli*, Staphylococci (coagulase-negative *Staphylococcus* spp., *S. aureus, and Staphylococcus* spp.) and *E. faecalis* isolated from livestock in a university veterinary diagnostic laboratory in Peninsular Malaysia. Significantly higher proportions of resistance among *E. coli* isolates were observed from non-ruminants compared to ruminants. However, the level of MDR did not significantly differ between the two groups. The findings indicate that the wide use of antibiotics, especially in non-ruminants for intensive production is linked to the higher resistance to various antibiotics. This is a worrying trend. More critical research is needed to investigate the patterns of resistance to antibiotics in food animals to ensure food safety.

## Data Availability Statement

The original contributions presented in the study are included in the article/supplementary material, further inquiries can be directed to the corresponding author/s.

## Author Contributions

NAH executed the laboratory work, collected and analyzed the data, and wrote the manuscript. LH devised the project design and main conceptual ideas, facilitated data analyses and interpretation of data, and co-write the manuscript. SKB and NIA facilitated the laboratory technical work and manuscript conception. SMJ facilitated data analysis and interpretation of findings. All authors have read and agreed to the published version of the manuscript.

## Conflict of Interest

The authors declare that the research was conducted in the absence of any commercial or financial relationships that could be construed as a potential conflict of interest.

## References

[1] World Organization for Animal Health. OIE List of Antimicrobials of Veterinary Importance (2007). Available online at: https://www.oie.int/fileadmin/Home/eng/Our_scientific_expertise/docs/pdf/AMR/A_OIE_List_antimicrobials_May2018.pdf (accessed September 12, 2020).

[2] Food Agriculture Organization/World Health Organization/World Organization for Animal Health. Report of the FAO/WHO/OIE Expert Meeting FAO. In: FAO/WHO/OIE Stakeholders Meeting on Critically Important Antimicrobials. (2008). Available online at: http://www.fao.org/3/a-i0204e.pdf (accessed September 12, 2020).

[3] Graham JP Boland JJ Silbergeld E. Growth promoting antibiotics in food animal production: an economic analysis. Public Health Rep. (2007) 122:79–87. 10.1177/003335490712200111 17236612 PMC1804117

[4] Chattopadhyay MK. Use of antibiotics as feed additives: a burning question. Front Microbiol. (2014) 5:334. 10.3389/fmicb.2014.00334 25071747 PMC4078264

[5] FAAIR Scientific Advisory Panel. A Report of the Facts about Antibiotics in Animals and the Impact on Resistance (FAAIR) Project. The Need to Improve Antimicrobial Use in Agriculture Ecological and Human (2002). Available online at: https://www.iatp.org/sites/default/files/64_2_36922.pdf (accessed September 13, 2020).

[6] Scott AM Beller E Glasziou P Clark J Ranakusuma RW Byambasuren O. Is antimicrobial administration to food animals a direct threat to human health? A rapid systematic review. Int J Antimicrob Ag. (2018) 52:316–23. 10.1016/j.ijantimicag.2018.04.005 29660406

[7] O'Neill J. Review on Antimicrobial Resistance. Antimicrobial Resistance: Tackling a Crisis for the Health and Wealth of Nations. (2014). Available online at: https://amr-review.org/sites/default/files/160525_Final%20paper_with%20cover.pdf (accessed September 12, 2020).

[8] Robinson TP Bu DP Carrique-Mas J Fèvre EM Gilbert M Grace D. Antibiotic resistance is the quintessential one health issue. T Roy Soc Trop Med H. (2016) 110:377–80. 10.1093/trstmh/trw048 27475987 PMC4975175

[9] Sharma C Rokana N Chandra M Singh BP Gulhane RD Gill JPS. Antimicrobial resistance: its surveillance, impact, and alternative management strategies in dairy animals. Front Vet Sci. (2018) 4:237. 10.3389/fvets.2017.00237 29359135 PMC5766636

[10] Doyle ME. Multidrug-resistant pathogens in the food supply. Foodborne Pathog Dis. (2015) 12:261–79. 10.1089/fpd.2014.1865 25621383

[11] Brennan E Martins M McCusker MP Wang J Martins Alves B Hurley D. Multidrug-resistant *Escherichia coli* in bovine animals, Europe. Emerg Infect Dis. (2016) 22:1650. 10.3201/eid2209.160140 27533105 PMC4994333

[12] Harada K Shimizu T Mukai Y Kuwajima K Sato T Usui M. Phenotypic and molecular characterization of antimicrobial resistance in *Klebsiella* spp. Isolates from companion animals in Japan: clonal dissemination of multidrug-resistant extended-spectrum β-lactamase-producing *Klebsiella pneumoniae*. Front Microbiol. (2016) 7:1021. 10.3389/fmicb.2016.01021 27446056 PMC4925667

[13] Friese A Schulz J Laube H von Salviati C Hartung J Roesler U. Faecal occurrence and emissions of livestock-associated methicillin-resistant *Staphylococcus aureus* (laMRSA) and ESbl/AmpC-producing *E. coli* from animal farms in Germany. Berl Munch Tierarztl. (2013) 126:175–80. 10.2376/0005-9366-126-175 23540202

[14] Schmithausen RM Schulze-Geisthoevel SV Stemmer F El-Jade M Reif M Hack S. Analysis of transmission of MRSA and ESBL-E among pigs and farm personnel. PLoS ONE. (2015) 10:e0138173. 10.1371/journal.pone.0138173 26422606 PMC4589321

[15] Schmithausen RM Schulze-Geisthoevel SV Heinemann C Bierbaum G Exner M Petersen B. Reservoirs and transmission pathways of resistant indicator bacteria in the biotope pig stable and along the food chain: a review from a one health perspective. Sustainability. (2018) 10:3967. 10.3390/su10113967

[16] Gahamanyi N Mboera LEG Matee MI Mutangana D Komba EVG. Prevalence, risk factors, and antimicrobial resistance profiles of thermophilic *campylobacter* species in humans and animals in sub-saharan Africa: a systematic review. Int J Microbiol. (2020) 2020:1–12. 10.1155/2020/2092478 32025233 PMC6983289

[17] Grant A Hashem F Parveen S. Salmonella and campylobacter: antimicrobial resistance and bacteriophage control in poultry. Food Microbiol. (2016) 53:104–9. 10.1016/j.fm.2015.09.008 26678136

[18] Nyachuba DG. Foodborne illness: is it on the rise? Nutr Rev. (2010) 68:257–69. 10.1111/j.1753-4887.2010.00286.x 20500787

[19] Hernández-Cortez C Palma-Martínez I Gonzalez-Avila LU Guerrero-Mandujano A Solís RC Castro-Escarpulli G. Food poisoning caused by bacteria (food toxins). In: Poisoning - from Specific Toxic Agents to Novel Rapid and Simplified Techniques for Analysis. (2017) 33:1–40. 10.5772/intechopen.69953

[20] Ünüvar S. Microbial foodborne diseases. In: Foodborne Diseases. Malatya: Elsevier; Inönü University (2018). p. 1–31. 10.1016/B978-0-12-811444-5.00001-4

[21] Bager F Bortolaia V Ellis-Iversen J Hendriksen RS Høg BB Jensen LB. DANMAP 2015 - use of antimicrobial agents and occurence of antimicrobial resistance in bacteria from food animals, food and humans in Denmark. DANMAP. (2016) 2015:1–145. Available online at: https://backend.orbit.dtu.dk/ws/files/127669665/DANMAP_2015.pdf (accessed September 12, 2020).

[22] Bortolaia V Hendriksen RS Høg BB Ellis-Iversen J Korsgaard HB Petersen CK. DANMAP 2018 - use of antimicrobial agents and occurence of antimicrobial resistance in bacteria from food animals, food and humans in Denmark. DANMAP. (2019) 2018:1–177. Available online at: https://backend.orbit.dtu.dk/ws/files/210256829/DANMAP_2018_1.pdf (accessed September 12, 2020).

[23] European Centre for Disease Prevention and Control. Surveillance of Antimicrobial Resistance in Europe Annual Report of the European Antimicrobial Resistance Surveillance Network (EARS-Net) 2017. ECDC: Surveillance Report (2018). Available online at: https://www.ecdc.europa.eu/sites/default/files/documents/AMR%202017_Cover%2BInner-web_v3.pdf (accessed September 15, 2020).

[24] Ministry of Health Malaysia. Malaysian Action Plan on Antimicrobial Resistance (MyAP-AMR) 2017-2021. Ministry of Health Malaysia (2017). Available online at: https://rr-asia.oie.int/wp-content/uploads/2020/03/malaysia_malaysia-national_action_plan_-_final_29_june.pdf (accessed September 16, 2020).

[25] Jang SS Biberstein EL Hirsh DC. A Diagnostic: Manual of Veterinary Clinical Bacteriology and Mycology. Iowa: University of California Davis County (2008).

[26] Clinical Laboratory Standards Institute. Performance Standards for Antimicrobial Disk and Dilution Susceptibility Tests for Bacteria Isolated from Animals: Approved Standard-Fourth Edition. CLSI Document VET01-A4. Clinical Laboratory Standards Institute, Wayne, PA (and Performance Standards for Antimicrobial Disk and Dilution Susceptibility Tests for Bacteria Isolated from Animals: Second Informational Supplement. CLSI Document VET01-S2. Clinical Laboratory Standards Institute, Wayne, PS) (2015).

[27] Agarwal A Kapila K Kumar S. WHONET software for the surveillance of antimicrobial susceptibility. Med J Armed Forces India. (2009) 65:264–66. 10.1016/S0377-1237(09)80020-8 27408261 PMC4921382

[28] World Health Organization. WHONET Tutorial Data Analysis 1 for the Surveillance of Antimicrobial Resistance (2006). Available online at: https://whonet.org/Docs/WHONET%204.Data%20analysis%201.doc (accessed September 16, 2020).

[29] World Health Organization. WHONET Tutorial Data Analysis 2 for the Surveillance of Antimicrobial Resistance (2006). Available online at: https://whonet.org/Docs/WHONET%205.Data%20analysis%202.doc (accessed September 16, 2020).

[30] World Health Organization. Critically Important Antimicrobials for Human Medicine (2011). Available online at: https://apps.who.int/iris/bitstream/handle/10665/77376/9789241504485_eng.pdf;jsessionid=47C90DD440D5FEC79E43AA8F4D099E98?sequence=1 (accessed September 16, 2020).

[31] Shahaza O Mohd. Azizul O Zakirah S Muhammad Azim FA Syamsyul A Maswati MA. Antimicrobial resistance in veterinary clinical isolates of *Escherichia coli* from Northern Region of Peninsular Malaysia. Malays J Vet Res. (2017) 8:1–7. Available online at: http://www.dvs.gov.my/dvs/resources/user_15/MJVR%20Volume%208%20No.1%202017/MJVR8N2/MJVR-V8N2-p1-7.pdf (accessed September 12, 2020).

[32] Singh F Sonawane GG Kumar J Dixit SK Meena RK Tripathi BN. Antimicrobial resistance and phenotypic and molecular detection of extended-spectrum β-lactamases among extraintestinal *Escherichia coli* isolated from pneumonic and septicemic sheep and goats in Rajasthan, India. Turk J Vet Anim Sci. (2019) 43:754–60. 10.3906/vet-1905-1

[33] Yassin AK Gong J Kelly P Lu G Guardabassi L Wei L. Antimicrobial resistance in clinical *Escherichia coli* isolates from poultry and livestock, China. PLoS ONE. (2017) 12:e0185326. 10.1371/journal.pone.0185326 28934348 PMC5608385

[34] Saidi R Mimoune N Baazizi R Benaissa MH Khelef D Kaidi R. Antibiotic susceptibility of *Staphylococci* isolated from bovine mastitis in Algeria. J Adv Vet Anim Res. (2019) 6:231. 10.5455/javar.2019.f337 31453196 PMC6702884

[35] Turutoglu H Ercelik S Ozturk D. Antibiotic resistance of *Staphylococcus aureus* and coagulase-negative *Staphylococci* isolated from Bovine Mastitis. Bull Vet Inst Pulawy. (2006) 50:41–45. Available online at: https://www.researchgate.net/publication/221670171_Antibiotic_resistance_of_Staphylococcus_aureus_and_coagulase-negative_staphylococci_isolated_from_bovine_mastitis (accessed September 12, 2020).

[36] Phophi L Petzer IM Qekwana DN. Antimicrobial resistance patterns and biofilm formation of coagulase-negative *Staphylococcus* species isolated from subclinical mastitis cow milk samples submitted to the onderstepoort milk laboratory. BMC Vet Res. (2019) 15:420. 10.1186/s12917-019-2175-3 31771575 PMC6880574

[37] Abo-Shama. Prevalence and Antimicrobial Susceptibility of *Staphylococcus aureus* Isolated from Cattle, Buffalo, Sheep and Goat's Raws Milk in Sohag Governorate, Egypt. Assiut Vet Med J. (2014) 60:63–72. Available online at: http://www.aun.edu.eg/journal_files/160_J_5513.pdf (accessed September 12, 2020).

[38] Kunz F Corti S Giezendanner N Stephan R Wittenbrink MM Zweifel C. Antimicrobial resistance of *Staphylococcus aureus* and coagulase-negative *Staphylococci* isolated from mastitis milk samples from sheep and goats. Schweiz Arch Tierheilkd. (2011) 153:63–9. 10.1024/0036-7281/a000152 21274832

[39] Martins T Rosa AF Castelani L de Miranda MS Arcaro JRP Pozzi CR. Intramammary treatment with gentamicin in lactating cows with clinical and subclinical mastitis. Pesqui Vet Bras. (2016) 36:283–9. 10.1590/S0100-736X2016000400006

[40] Erskine RJ Walker RD Bolin CA Bartlett PC White DG. Trends in antibacterial susceptibility of mastitis pathogens during a seven-year period. J Dairy Sci. (2002) 85:1111–8. 10.3168/jds.S0022-0302(02)74172-6 12086045

[41] Niamir-Fuller M. Towards sustainability in the extensive and intensive livestock sectors. OIE Rev Sci Tech. (2016) 35:371–87. 10.20506/rst.35.2.2531 27917985

[42] Marti E Variatza E Balcázar JL. Bacteriophages as a Reservoir of extended-spectrum β-lactamase and Fluoroquinolone Resistance Genes in the Environment. Clin Microbiol Infect. (2014) 20:O456–9. 10.1111/1469-0691.12446 24552593

[43] Reddy B Dubey SK. River Ganges Water as Reservoir of Microbes with Antibiotic and Metal Ion Resistance Genes: High Throughput Metagenomic Approach. Environ Pollut. (2019) 246:443–51. 10.1016/j.envpol.2018.12.022 30579213

[44] Jajere SM Hassan L Zakaria Z Abu J Aziz SA. Antibiogram profiles and risk factors for multidrug resistance of *Salmonella enterica* recovered from village chickens (*Gallus gallus domesticus linnaeus*) and other environmental sources in the Central and Southern Peninsular Malaysia. Antibiot. (2020) 9:701. 10.3390/antibiotics9100701 33076451 PMC7602575

[45] Dosen R Prodanov-Radulovic J Pusic I Stojanov I Stojanovic D Ratajac R. Resistance *Escherichia coli* isolates to antibiotics from the organ samples originating from Swine Farms. Biotechnol Anim Husb. (2011) 27:861–6. 10.2298/BAH1103861D

[46] Kazemnia A Ahmadi M Dilmaghani M. Antibiotic resistance pattern of different *escherichia coli* phylogenetic groups isolated from human urinary tract infection and avian colibacillosis. Iran Biomed J. (2014) 18:219. 10.6091/ibj.1394.2014 25326020 PMC4225061

[47] Rahman MA Rahman Akma Islam MA Alam MM. Antimicrobial resistance of *escherichia coli* isolated from milk, beef and chicken meat in Bangladesh. Bangladesh J Vet Med. (2017) 15:141–6. 10.3329/bjvm.v15i2.35525

[48] Karczmarczyk M Abbott Y Walsh C Leonard N Fanning S. Characterization of multidrug-resistant *Escherichia coli* isolates from animals presenting at a University Veterinary Hospital. Appl Environ Microbiol. (2011) 77:7104–12. 10.1128/AEM.00599-11 21856835 PMC3194860

[49] Perrin-Guyomard A Jouy E Urban D Chauvin C Granier SA Mourand G. Decrease in fluoroquinolone use in french poultry and pig production and changes in resistance among *E. coli* and Campylobacter. Vet Microbiol. (2020) 4:108637. 10.1016/j.vetmic.2020.108637 32273016

[50] The European Medicines Agency. Committee for Veterinary Medicinal Products. Enrofloxacin (Summary Report 1) (1998). Available online at: https://www.ema.europa.eu/en/documents/mrl-report/enrofloxacin-extension-all-food-producing-species-summary-report-5-committee-veterinary-medicinal_en.pdf (accessed September 18, 2020).

[51] Gouvêa R Dos Santos FF De Aquino MH. Fluoroquinolones in industrial poultry production, bacterial resistance and food residues: a review. Rev Bras Cienc Avic. (2015) 17:1–10. 10.1590/1516-635x17011-1029537307

[52] Sárközy G. Quinolones: a class of antimicrobial agents. Vet Med. (2001) 46:257–74. 10.17221/7883-VETMED

[53] Liu XQ Wang J Li W Zhao LQ Lu Y Liu JH. Distribution of Cfr in *Staphylococcus* spp. and *Escherichia coli* Strains from Pig Farms in China and Characterization of a Novel Cfr-carrying F43: A-: B- Plasmid. Front Microbiol. (2017) 8:329. 10.3389/fmicb.2017.00329 28293235 PMC5329041

[54] Noh EB Kim YB Seo KW Son SH Ha JS Lee YJ. Antimicrobial resistance monitoring of commensal *Enterococcus faecalis* in broiler breeders. Poulty Sci. (2020) 99:2675–83 10.1016/j.psj.2020.01.014 32359604 PMC7597544

[55] De Graef EM Decostere A Devriese LA Haesebrouck F. Antibiotic resistance among fecal indicator bacteria from healthy individually owned and kennel dogs. Microb Drug Resist. (2004) 10:65–9. 10.1089/107662904323047826 15140396

[56] Aarestrup FM Wegener HC Collignon P. Resistance in bacteria of the food chain: epidemiology and control strategies. Expert Rev Anti Infect The. (2008) 6:733–50. 10.2217/ebo.12.361 18847409

[57] Aasmäe B Häkkinen L Kaart T Kalmus P. Antimicrobial resistance of *Escherichia coli* and *Enterococcus* spp. Isolated from Estonian Cattle and Swine from 2010 to 2015. Acta Vet Scand. (2019) 61:5. 10.1186/s13028-019-0441-9 30665443 PMC6341677

[58] Abbassi MS Kilani H Zouari M Mansouri R Oussama EF. Antimicrobial resistance in *Escherichia coli* isolates from healthy poultry, bovine and ovine in Tunisia: a real animal and human health threat. J Clin Microbiol Biochem Technol. (2017) 3:019–123. 10.17352/jcmbt.000021

[59] Enne VI Cassar C Sprigings K Woodward MJ Bennett PM. A high prevalence of antimicrobial resistant *Escherichia coli* isolated from pigs and a low prevalence of antimicrobial resistant *E. coli* from Cattle and Sheep in Great Britain at Slaughter. FEMS Microbiol Lett. (2008) 278:193–9. 10.1111/j.1574-6968.2007.00991.x 18053066

[60] Lei T Tian W He L Huang XH Sun YX Deng YT. Antimicrobial resistance in *Escherichia coli* isolates from food animals, animal food products and companion animals in China. Vet Microbiol. (2010) 146:85–9. 10.1016/j.vetmic.2010.04.025 20605690

[61] Jiang HX Lü DH Chen ZL Wang XM Chen JR Liu YH. High prevalence and widespread distribution of multi-resistant *Escherichia coli* isolates in pigs and poultry in China. Vet J. (2011) 187:99–103. 10.1016/j.tvjl.2009.10.017 19926317

[62] Boireau C Cazeau G Jarrige N Calavas D Madec JY Leblond A. Antimicrobial resistance in bacteria isolated from mastitis in dairy cattle in France, 2006– 2016. J Dairy Sci. (2018) 101:9451–62. 10.3168/jds.2018-14835 30100506

[63] Sayah RS Kaneene JB Johnson Y Miller R. Patterns of antimicrobial resistance observed in *Escherichia coli* isolates obtained from domestic- and wild-animal fecal samples, human septage, and surface water. Appl Environ Microbiol. (2005) 71:1394–404. 10.1128/AEM.71.3.1394-1404.2005 15746342 PMC1065171

[64] Carrique-Mas JJ Choisy M Van Cuong N Thwaites G Baker S. An estimation of total antimicrobial usage in humans and animals in Vietnam. Antimicrob Resist Infect Control. (2020) 9:1–6. 10.1186/s13756-019-0671-7 31956405 PMC6961235

[65] Van Boeckel TP Brower C Gilbert M Grenfell BT Levin SA Robinson TP. Global trends in antimicrobial use in food animals. Proc Natl Acad Sci USA. (2015) 112:5649–54. 10.1073/pnas.1503141112 25792457 PMC4426470

[66] Organisation for Economic Co-operation and Development. Global Antimicrobial Use in the Livestock Sector. In: Working Party on Agricultural Policies and Markets (2015). Available online at: www.oecd.org/officialdocuments/publicdisplaydocumentpdf/?cote=TAD/CA/APM/WP%282014%2934/FINAL&docLanguage=En (accessed September 21, 2020).

[67] Hassali MA Yann HR Verma AK Hussain R Sivaraman S. Antibiotic use in food animals : Malaysia overview. Discipline of Social & Administrative Pharmacy. School of Pharmaceutical Sciences. Universiti Sains Malaysia. (2018) 11800.

[68] Lerminiaux NA Cameron AD. Horizontal transfer of antibiotic resistance genes in clinical environments. Can J Microbiol. (2019) 65:34–44. 10.1139/cjm-2018-0275 30248271

[69] Sørensen SJ Bailey M Hansen LH Kroer N Wuertz S. Studying plasmid horizontal transfer *in situ*: a critical review. Nat Rev Microbiol. (2005) 3:700–10. 10.1038/nrmicro1232 16138098

[70] Szmolka A Nagy B. Multidrug resistant commensal *escherichia coli* in animals and its impact for public health. Front Microbiol. (2013) 4:258. 10.3389/fmicb.2013.00258 24027562 PMC3759790

[71] Blair JMA Webber MA Baylay AJ Ogbolu DO Piddock LJV. Molecular mechanisms of antibiotic resistance. Nat Rev Microbiol. (2015) 13:42–51. 10.1038/nrmicro3380 25435309

